# Optic Nerve Metastasis in Lung Adenocarcinoma

**DOI:** 10.7759/cureus.55413

**Published:** 2024-03-02

**Authors:** Yelumalai Mangalathevi, Yaakub Azhany, Wan-Hazabbah Wan Hitam, Mohd Ezane Aziz

**Affiliations:** 1 Department of Ophthalmology and Visual Science, School of Medical Sciences, Health Campus, Universiti Sains Malaysia, Kelantan, MYS; 2 Hospital Universiti Sains Malaysia, Health Campus, Universiti Sains Malaysia, Kelantan, MYS; 3 Department of Radiology, School of Medical Sciences, Health Campus, Universiti Sains Malaysia, Kelantan, MYS

**Keywords:** ocular metastasis, optic nerve infiltration, adenocarcinoma, lung cancer, optic nerve

## Abstract

In this article, we report a rare case of an optic nerve metastasis secondary to lung adenocarcinoma. The ocular manifestation was the first clinical sign of the disease, and further investigation led to the diagnosis of the underlying malignancy. A 59-year-old woman presented with progressive blurring of vision in the right eye for the past month. She had been having headaches for the past two weeks and left upper limb weakness for one day. She also had loss of appetite and weight for the past few months. She looked lethargic. On presentation, her bilateral eye vision was 6/18. Both anterior segments were unremarkable. Fundoscopy showed a normal optic disc in both eyes. A nervous system examination showed mild motor sensory impairment over the left upper and lower limbs and also impairment of cranial nerves V and VII. Brain computed tomography was conducted and revealed soft tissue lesions at the lateral aspect of the optic nerve and multiple recent cerebral infarcts. Brain and orbital magnetic resonance imaging showed a metastasis intraconal lesion at the right intraorbital segment of the optic nerve. CT thorax, abdomen, and pelvis were done. The finding revealed carcinoma of the left lung with distant metastasis. The patient’s general condition deteriorated in less than two weeks. The family refused further intervention. The patient died three months after the initial presentation.

## Introduction

Metastasis involving ocular structures is rare [1]. Ocular metastasis incidence has been reported to vary from 0.07 to 12% [2]. The choroid, which has an ample blood supply, is a common site for ocular metastasis, which mostly originates from the breast and lung [3]. Optic nerve metastasis is very rare as only 4.5% of all ocular metastasis cases have been reported [4]. Women usually present with metastatic malignancy involving the eye arising from breast cancer and in men from lung cancer [1]. Lung cancer is the leading cause of cancer-related death in Malaysia [5]. Here, we report a rare case of an optic nerve metastasis from lung adenocarcinoma in a woman.

## Case presentation

A 59-year-old female presented to the emergency department with right eye blurred vision for the past one month. She had generalized blurring of vision, which was painless and progressively worsening for one month. She also had left-side body weakness for one day. Family members noticed she has been having difficulty performing her daily routine activities using her left hand. She was dropping objects and had difficulty lifting her left arm. She complained of occasional early morning headaches which radiated from the frontal to the occipital region for the past month. She also had constitutional symptoms such as loss of appetite, weight loss, and fatigue for the past few months. She had no other systemic symptoms such as cough, fever, or shortness of breath. There was no significant medical history documented. She was a nonsmoker. Her father died at the age of 69 due to lung cancer.

At the presentation, her bilateral eye vision was 6/18. Both pupillary reactions and optic nerve functions were normal. Extraocular mobility was full in both eyes. The anterior segment findings were unremarkable. The intraocular pressure was within normal limits in both eyes. Fundoscopy showed a normal optic disc in both eyes. On systemic examination, there was a presence of weakness (motor power 4/5) and reduced sensation in the left upper and lower limbs. There was also the presence of trigeminal and facial nerve impairments. She had loss of left nasolabial fold and reduced sensation over her face. Therefore, she was investigated further with tumor markers and imaging. Her tumor markers, namely, carcinoembryonic antigen (CEA) and CA -125, were markedly raised.

**Figure 1:**
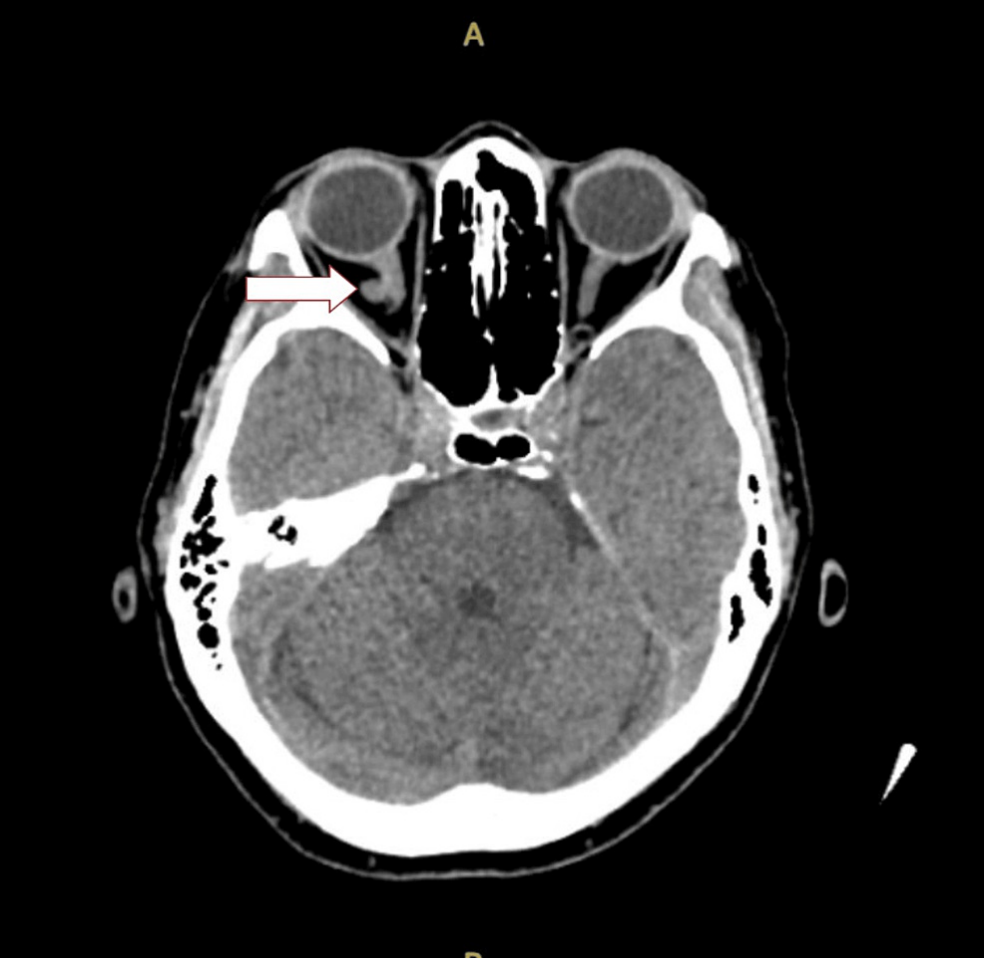
Plain CT brain shows a pedunculated soft tissue lesion at the lateral aspect of right optic nerve measuring 0.5 x 0.5 x 0.5 cm (AP x W X CC)

**Figure 2:**
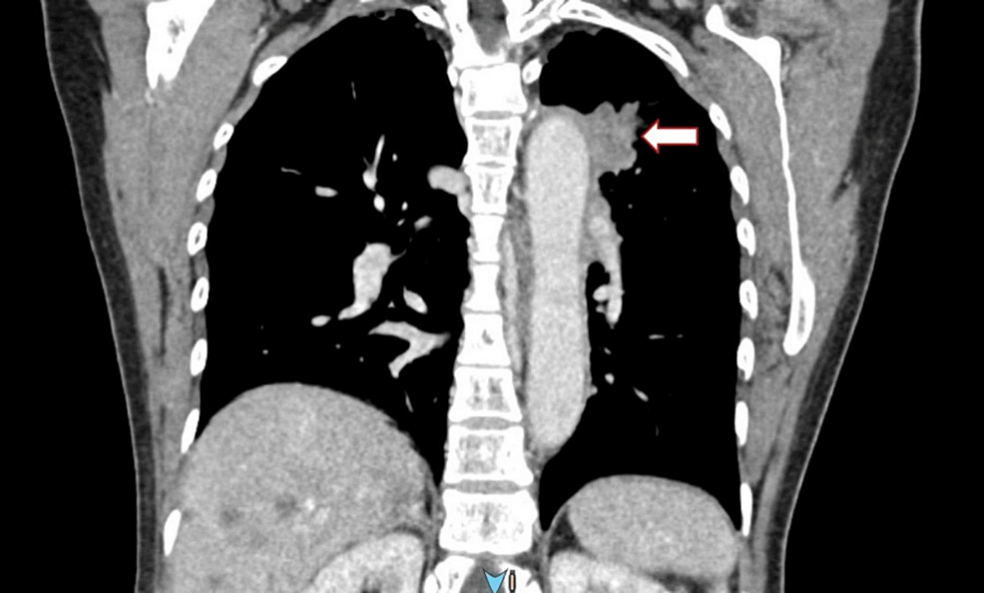
CT thorax showing irregularly shaped mass in the apicoposterior segment apical of the left upper lobe measuring 4.6 x 3.0x 1.9 cm (length x width x AP)

**Figure 3:**
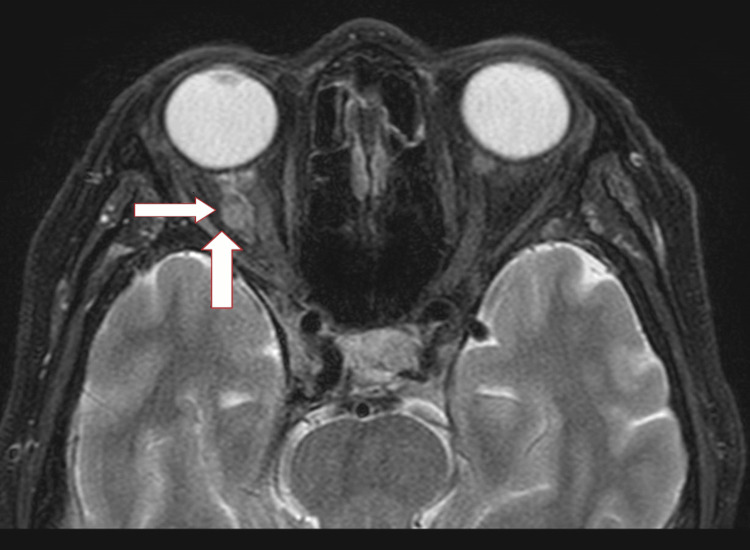
MRI orbits show a well-defined intraconal lesion measuring 0.5 x 0.5 x 0.5 cm (AP X W X CC) at the lateral aspect of the right optic nerve in the intraorbital segment. The lesion was displacing the right optic nerve medially.

A brain computed tomography scan showed multiple right occipital recent infarcts and pedunculated soft tissue lesions at the lateral aspect of the right optic nerve measuring 0.5 x 0.5 x 0.5 cm (AP X W X CC) (Figure 1). Further imaging and computed tomography were performed over the thorax, abdomen, and pelvis. A scan revealed carcinoma of the left lung (upper lobe) with lymph nodes, liver, spleen, right adrenal, and bone metastasis (Figure 2). Brain and orbital magnetic resonance imaging showed multiple lesions in the bilateral cerebrum and cerebellum, which suggested distant metastasis. A well-defined intraconal lesion at the lateral aspect of the right optic nerve in the intraorbital segment represented metastasis (Figure 3). The lesion showed heterogenous enhancement with dural enhancement postcontrast. The lesion was displacing the right optic nerve medially. Lung bronchoscopy (bronchoalveolar lavage) and cytology revealed lung adenocarcinomas.

The patient was admitted to the ward for monitoring. Her optic nerve functions deteriorated in less than two weeks. The relative afferent pupillary defect was detected in the right eye. Her light brightness and red saturation were reduced. The visual field of confrontation and color plates suggested a deterioration of optic nerve function. There was the presence of left homonymous hemianopia in the visual field test. The bilateral eye cup disc ratio was 0.7, and the rim appeared healthy. However, there was no lesion on the choroid or retina. Treatment was planned for the patient, but she defaulted on her follow-up. The patient died three months after diagnosis.

## Discussion

Eye metastasis generally originates from lung cancer (21 - 24%) and breast cancer (28.5 - 58.8%) [6,7]. Lung cancer generally causes hematogenous metastasis due to blood-brain barrier disruption [6]. Ocular metastasis occurs in the choroid (88%), iris (9%), ciliary body (2%), and retina (less than 1%) [8]. Optic nerve metastasis is not common, and it is usually an extension from the choroid, orbit, or central nervous system [9]. The case series involving 227 patients with ocular metastases done by Ferry and Font reported metastatic optic nerve compromise in only three cases (1.3%). The sites of primary tumors in metastatic cases are mostly located in the breast and lung, which are 39.7% and 29.5%, respectively [10]. Shankar et al. reported that lung malignancy accounts for 8 to 12% of orbital metastasis [11].

Commonly, eye metastasis patients present with visual impairment, double vision, proptosis, eyelid swelling, limited ocular motility, pain, eye redness, floaters, and a visual field defect [10-12]. Pain is usually not a chief complaint unless there is an extensive intraocular tumor [1]. Hernandez et al. reported a case of isolated optic nerve metastasis in patients with lung adenocarcinoma who presented with a sudden loss of visual acuity [9]. Our patient’s first manifestation was painless visual impairment prior to the diagnosis of an underlying malignancy, which may have been misdiagnosed as the patient also had stroke symptoms. Similarly, Lai et al. reported a case of visual impairment as an initial presentation prior to the diagnosis of lung cancer in a patient [8]. Tamai et al. also documented a similar case where the patient initially presented with visual impairment prior to the diagnosis of primary malignancy [13].

Imaging plays an important role in the diagnosis of metastatic lesions. Other options are available, such as fine needle aspiration and pathological evaluation. In our case, the patient's fundoscopy was normal; however, imaging of the brain and orbit showed an underlying illness. B-scan ocular ultrasounds can be used as well, if available [1].

Management of eye metastasis involves multiple disciplines such as ophthalmologists, oncologists, and radiation therapists. There are several treatment options available such as observation, chemotherapy, photocoagulation, cryotherapy, surgical resection, and radiotherapy [1]. The treatment of choice will be based on patient staging, histopathological analysis of primary malignancy, and clinical condition. A symptomatic patient requires prompt treatment as soon as the diagnosis is established. Lung cancer is radio-sensitive and treated with low-dose external beam radiation [1]. In our case, the patient and family members were not keen on further intervention. It is effective to treat chemo-sensitive tumors, such as small-cell lung cancer, with systemic chemotherapy. Metastatic cancer will be progressive if left untreated, and optic nerve metastasis leads to rapid vision loss [1].

Eye metastasis accounts for a poor prognosis, with median survival times ranging from three months to one year [11]. Cherekaev et al. reported an unfavorable prognosis for survival and visual acuity in breast carcinoma metastatic to the eye [14]. Similarly, Biswas et al. showed that metastatic involvement of the optic disc has a poor visual prognosis [15]. Lampaki et al. further found that ocular involvement in metastatic tumors has poor prognostic signs for long-term survival [1]. In our case, the patient succumbed to death due to the progression of the disease within three months of the initial presentation.

## Conclusions

Although ocular manifestation is very rare for lung carcinoma, further investigations must be conducted in the presence of systemic and nervous system involvement. Early diagnosis may be complicated by nonspecific clinical features; however, imaging should be considered in highly suspect patients. In our case, a detailed clinical history and further investigation led to the diagnosis of the underlying malignancy.
